# Adherence to CDC Recommendations for the Treatment of Uncomplicated Gonorrhea — STD Surveillance Network, United States, 2016

**DOI:** 10.15585/mmwr.mm6716a4

**Published:** 2018-04-27

**Authors:** Emily J. Weston, Kimberly Workowski, Elizabeth Torrone, Hillard Weinstock, Mark R. Stenger

**Affiliations:** ^1^Division of STD Prevention, National Center for HIV/AIDS, Viral Hepatitis, STD and TB Prevention, CDC; ^2^Department of Medicine, Emory University, Atlanta, GA.

Gonorrhea, the sexually transmitted disease (STD) caused by *Neisseria gonorrhoeae*, is the second most common notifiable disease in the United States after chlamydia; 468,514 cases were reported to state and local health departments in 2016, an increase of 18.5% from 2015 (*1*). *N. gonorrhoeae* has progressively developed resistance to most antimicrobials used to treat the infection (*2*). As a result, CDC recommends two antimicrobials (250 mg of ceftriaxone [IM] plus 1 g of azithromycin [PO]) for treating uncomplicated gonorrhea to improve treatment efficacy and, potentially, to slow the emergence and spread of antimicrobial resistance. To monitor adherence to the current CDC-recommended regimen for uncomplicated gonorrhea, CDC reviewed enhanced data collected on a random sample of reported cases of gonorrhea in seven jurisdictions participating in the STD Surveillance Network (SSuN) and estimated the proportion of patients who received the CDC-recommended regimen for uncomplicated gonorrhea, by patient characteristics and diagnosing facility type. In 2016, the majority of reported patients with gonorrhea (81%) received the recommended regimen. There were no differences in the proportion of patients receiving the recommended regimen by age or race/ethnicity; however, patients diagnosed with gonorrhea in STD (91%) or family planning/reproductive health (94%) clinics were more likely to receive this regimen than were patients diagnosed in other provider settings (80%). These data document high provider adherence to CDC gonorrhea treatment recommendations in specialty STD clinics, indicating high quality of care provided in those settings. Local and state health departments should monitor adherence with recommendations in their jurisdictions and consider implementing interventions to improve provider and patient compliance with gonorrhea treatment recommendations where indicated.

SSuN is a CDC-supported, sentinel surveillance project comprised of 10 selected state and city health departments that conduct investigations to collect supplementary information on a random sample of gonorrhea cases reported from all health care providers/reporting sources in their jurisdictions (https://www.cdc.gov/std/ssun/default.htm). These investigations include contacting the diagnosing provider to verify treatment and conducting patient interviews to collect behavioral and demographic information. Case weights were developed to account for local sample fractions and adjust for nonresponse by patient sex and age group, allowing CDC to produce estimates representative of all reported gonorrhea cases in these jurisdictions (*3*).

Analyses were restricted to seven of the 10 SSuN jurisdictions (Baltimore, Maryland; California, excluding San Francisco; Florida; Massachusetts; Multnomah County, Oregon; Minnesota; and Philadelphia, Pennsylvania) with documented treatment information (antimicrobials and dosages) for ≥90% of cases with complete investigations. Cases with missing patient treatment information (6.7%) were excluded from further analysis. Based on provider report of treatment provided, patients treated with the recommended dual therapy for uncomplicated gonorrhea (i.e., 250 mg dose of ceftriaxone [IM] plus 1 g dose of azithromycin [PO]) were classified as having received the recommended regimen. All other patients were classified as having received other regimens. Weighted estimates of the number and proportion of patients treated with the recommended regimen and corresponding 95% confidence intervals (CI) were calculated. Prevalence ratios (PRs) were estimated to identify differences in documented treatment by patient characteristics and diagnosing facility type. Gay, bisexual, and other men who have sex with men (MSM) were defined as any male patient reporting male sex partners in the previous 2–3 months or reporting their sexual orientation as gay or bisexual.

In 2016, a total of 91,719 gonorrhea cases were reported in the seven participating SSuN jurisdictions. Among these, 8,393 (9.2%) were randomly sampled; complete provider investigations were obtained for 3,213 cases for a response rate of 38%. Overall, 93.3% of these patients had a treatment documented and were included in the analysis.

Based on weighted analysis,* CDC estimated that 81.3% (95% CI = 79.2–83.4) of reported patients with gonorrhea in these SSuN jurisdictions were treated with the recommended dual therapy for uncomplicated gonorrhea (Table 1). The percentage of patients treated with this regimen varied by jurisdiction (range = 76.7% to 92.0%). There were no differences by patient age or race/ethnicity (Table 2). Although not statistically significant, women were somewhat less likely than men to receive the recommended regimen (79.3% versus 82.5%; PR = 0.96, 95% CI = 0.91–1.02). MSM were more likely to receive the recommended regimen compared with heterosexual men and women (84.8% versus 79.4%; PR = 1.07, 95% CI = 1.01–1.13). Patients diagnosed with gonorrhea in family planning/reproductive health clinics were more likely to receive the recommended regimen than were patients diagnosed in other provider settings (93.8% versus 79.5%; PR = 1.18, 95% CI = 1.13–1.23). Similarly, patients diagnosed in STD clinics were more likely to receive the recommended regimen than were patients diagnosed in other provider settings (90.8% versus 79.8%; PR = 1.14, 95% CI = 1.08–1.20). When stratified by sexual behavior, patients whose gonorrhea was diagnosed in STD and family planning/reproductive health clinics were more likely to be treated with the recommended regimen whether or not they were MSM.

**TABLE 1:** Estimated number of gonorrhea cases by treatment regimens received — STD Surveillance Network, United States, 2016

Treatment regimen	Weighted no.*	Weighted % (95% CI)*
**Recommended treatment for uncomplicated gonorrhea**		
Ceftriaxone 250 mg + azithromycin 1 g	74,599	81.3 (79.2–83.4)
**Other regimens**		
Ceftriaxone 250 mg only	5,430	5.9 (4.8–7.0)
Ceftriaxone any dosage + doxycycline	4,016	4.4 (3.3–5.5)
Azithromycin only	2,884	3.1 (2.1–4.1)
Ceftriaxone + azithromycin (other or unknown dosage)	1,936	2.1 (1.3–2.9)
Doxycycline only	1,055	1.2 (0.5–1.8)
Cefixime + azithromycin or doxycycline	599	0.7 (0.5–0.9)
Ceftriaxone (125 mg or unknown dosage) only	530	0.6 (0.2–1.0)
Other antimicrobials^†^	420	0.5 (0.0–1.0)
Cefotaxime 1 g + azithromycin 1 g or ceftizoxime 1 g + azithromycin 1 g	115	0.1 (0.0–0.3)
Cefixime only	83	0.1 (0.0–0.2)
Azithromycin 2 g + gentamicin or gemifloxacin	51	0.1 (0–0.1)

**Abbreviations**: CI = confidence interval, STD = sexually transmitted disease.

* No., %, and 95% CI reflect weighted estimates for all reported gonorrhea cases; minor variance in weights might cause category estimates to total to slightly more or less than overall case estimate.

^†^ Other antimicrobials include azithromycin 1 g + doxycycline, fluoroquinolone alone, and gentamicin alone.

**TABLE 2:** Estimated cases by patient demographics, diagnosing facility type, and treatment regimen received — STD Surveillance Network, United States, 2016

Characteristic	Regimen received				Prevalence ratio (95% CI)
	Recommended for uncomplicated gonorrhea		Other		
	Weighted no.*	Weighted % (95% CI)*	Weighted no.*	Weighted % (95% CI)*	
**Total**	**74,599**	**81.3 (79.2–83.4)**	**17,120**	**18.7 (16.6–20.8)**	**—**
**Gender and sex of sex partner(s)**					
Women	26,088	79.3 (75.5–83.0)	6,822	20.7 (17.0–24.5)	0.96 (0.91–1.02)
MSM	27,804	84.8 (81.4–88.2)	4,994	15.2 (11.8–18.8)	1.07 (1.01–1.13)
MSW	18,641	78.9 (74.8–83.0)	4,993	21.1 (17.0–25.2)	0.96 (0.90–1.02)
Men with unknown sex of sex partner(s)	2,066	86.9 (79.7–94.1)	311	13.1 (5.9–20.3)	1.07 (0.98–1.17)
**Age group (yrs)**					
≤19	10,570	83.1 (77.5–88.7)	2,148	16.9 (11.3–22.5)	1.03 (0.95–1.10)
20–24	19,842	81.2 (77.2–85.3)	4,586	18.8 (14.7–22.8)	1.00 (0.94–1.06)
25–29	17,600	84.3 (80.1–88.5)	3,283	15.7 (11.5–19.9)	1.05 (0.99–1.11)
30–34	9,901	80.0 (74.4–85.7)	2,468	20.0 (14.3–25.6)	0.98 (0.91–1.06)
35–39	5,887	77.3 (69.1–85.5)	1,729	22.7 (14.5–30.9)	0.95 (0.85–1.06)
40–44	3,697	82.1 (72.3–91.9)	806	17.9 (8.1–27.7)	1.01 (0.89–1.14)
≥45	7,099	77.2 (70.2–84.1)	2,100	22.8 (15.9–29.8)	0.94 (0.86–1.04)
**Race/Ethnicity**					
White	16,424	77.4 (72.7–82.0)	4,808	22.6 (18.0–27.3)	0.94 (0.88–1.00)
Black	29,178	82.5 (79.5–85.6)	6,172	17.5 (14.4–20.5)	1.02 (0.97–1.08)
Hispanic	21,492	84.8 (80.8–88.8)	3,853	15.2 (11.2–19.2)	1.06 (1.00–1.12)
All other races	5,210	81.5 (72.4–90.6)	1,185	18.5 (9.4–27.6)	1.00 (0.89–1.12)
Missing/Refused	2,294	67.5 (54.5–80.6)	1,103	32.5 (19.4–45.5)	0.82 (0.68–1.00)
**Diagnosing provider/Facility type**					
STD clinic	11,565	90.8 (87.0–94.6)	1,174	9.2 (5.4–13.0)	1.14 (1.08–1.20)
Private provider/HMO/ PPO	19,090	75.8 (70.9–80.6)	6,104	24.2 (19.4–29.1)	0.91 (0.85–0.97)
Hospital ED/Emergent/Urgent care	3,249	74.6 (67.8−81.5)	1,105	25.4 (18.5–32.2)	0.91 (0.83–1.01)
Family planning/ Reproductive health clinics	11,319	93.8 (91.0–96.6)	748	6.2 (3.4–9.0)	1.18 (1.13–1.23)
Other HD/Public clinics or tribal clinics	4,516	88.1 (80.7–95.5)	610	11.9 (4.5–19.3)	1.09 (1.00–1.19)
All other^†^	10,506	86.3 (82.2–90.5)	1,665	13.7 (9.5–17.8)	1.07 (1.01–1.13)
Unknown	14,353	71.5 (66.0–77.1)	5,715	28.5 (22.9–34.0)	0.85 (0.78–0.92)

**Abbreviations**: CI = confidence interval; ED = emergency department; HD = health department; HIV = human immunodeficiency virus; HMO = health maintenance organization; MSM = men who have sex with men; MSW = men who have sex with women only; PPO = preferred provider organization; STD = sexually transmitted disease.

* No., %, and 95% CI reflect weighted estimates for all reported gonorrhea cases; minor variance in weights might cause category estimates to total to slightly more or less than overall case estimate.

^†^ All other includes: HIV primary/specialty care or HIV testing sites, correctional facilities, school-based pediatric or adolescent care, and other provider types.

Overall, 18.7% (95% CI = 16.6–20.8) of patients received other regimens (Table 1). The most frequent other regimens reported were ceftriaxone 250 mg only (5.9%), ceftriaxone any dosage and doxycycline (4.4%), and azithromycin only (3.1%). Fewer than 0.5% of patients were treated with a regimen suggesting treatment of a patient with a cephalosporin allergy (e.g., azithromycin plus either gentamicin or gemifloxacin) or a patient with a complicated gonococcal infection (e.g., azithromycin plus either cefotaxime or ceftizoxime).

## Discussion

CDC’s gonorrhea treatment recommendations are periodically revised based on the best available evidence of emerging trends in antimicrobial susceptibility. Provider awareness of, and adherence to current treatment recommendations helps ensure that all patients are treated with the most effective therapy and might decrease the development of antimicrobial resistance. Monitoring treatment practices across all provider and diagnostic settings helps identify opportunities for interventions to increase provider adherence. The current analysis provides estimates of treatment practices among all providers diagnosing gonococcal infections in seven of 10 SSuN jurisdictions and are the first published estimates of adherence to CDC recommendations since gonorrhea treatment guidelines were revised in 2012 and in 2015 (*2*,*4*).

This analysis documents high levels of compliance with CDC treatment recommendations, with 81% of patients receiving recommended dual therapy for uncomplicated gonorrhea and substantiate high levels of compliance observed in previous analyses of gonorrhea cases reported in jurisdictions participating in SSuN during 2006–2008 and 2010–2012 (*5*,*6*). Optimally, all patients diagnosed with uncomplicated gonorrhea should be treated with the recommended regimen to ensure effective treatment and to help forestall the emergence of antimicrobial resistance. However, in practice, many factors might influence provider’s adherence to the recommended regimen, including the availability of injectable medications at the time of treatment and patient-reported allergies. In the current analysis, patients diagnosed with gonorrhea in STD and family planning/reproductive health clinics were more likely to be treated with the recommended regimen than were patients diagnosed in other provider settings, similar to observations from earlier studies (*7*,*8*). Across all provider settings, MSM were more likely to be treated with the recommended regimen, and MSM were more likely than non-MSM to receive a diagnosis in STD clinics. However, in stratified analyses by sexual behavior and diagnosing facility type, STD clinics were still more likely to treat with the recommended regimen than were other provider types. Implementation of guidelines in other provider settings might be influenced by a smaller volume of patients with gonorrhea seeking care and services, as providers diagnosing fewer cases might be less familiar with current recommendations.

The majority of patients treated with other regimens were treated with only one antimicrobial, including 3% of all patients treated with azithromycin only and 1.2% with doxycycline alone. Azithromycin monotherapy is not recommended for treatment of gonococcal infections because of concerns about emerging resistance and case reports of treatment failures (*1*,*2*,*9*). In addition, tetracycline has not been recommended as treatment regimen for gonorrhea since the 1980s because of established chromosomally and plasmid-mediated resistance in the United States (*10*). These findings reinforce the imperative for state and local jurisdictions to identify provider settings where patients are receiving inadequate treatment. Additional training and education on the importance of adherence to treatment recommendations might increase the proportion of patients adequately treated and further delay the emergence of antimicrobial-resistant gonorrhea.

The findings in this report are subject to at least four limitations. First, findings are based on enhanced investigations conducted for a random sample of gonorrhea cases in seven jurisdictions; SSuN is not designed to be nationally representative although these jurisdictions reported approximately 20% of all gonorrhea cases in the United States in 2016. Second, although case weights were calculated to account for differing sample fractions across SSuN jurisdictions and for nonresponse, it is possible that unmeasured bias exists. CDC is unable to adjust these data for nonresponse by provider type because the complete distribution by provider type in the underlying population of cases is unknown. If providers who were less likely to treat patients with a recommended therapy were also less likely to respond to investigators, this analysis might overestimate the proportion of patients treated with the recommended regimen. Third, a small number of patients might have had allergies or other clinical scenarios that would have been appropriately treated with an alternative regimen; however, allergies and complications are not documented during SSuN investigations. Consequently, findings might underestimate the proportion of appropriately treated patients with gonorrhea. Finally, treatment information was missing for 6.7% of sampled cases; it is plausible that these patients were treated with the recommended regimen, but investigators were unable to document treatment at the time of the investigation.

Despite the high level of treatment adherence documented in this analysis, improving provider adherence to treatment recommendations for antibiotic use across the full spectrum of health care settings is an integral part of a comprehensive approach to combating the emergence of antimicrobial-resistant gonorrhea. State and local health departments should continue to work with the providers and patients to assure timely detection and treatment of gonorrhea according to current CDC treatment recommendations (*2*).

**Summary**

**What is already known about this topic?**

CDC’s treatment recommendations for gonorrhea were revised in 2012 and 2015 based on emerging antimicrobial resistance.

**What is added by this report?**

In 2016, 81% of gonorrhea cases in seven jurisdictions were treated with the recommended regimen for uncomplicated gonorrhea (250 mg dose of ceftriaxone [IM] plus 1 g dose of azithromycin [PO]), but this varied by provider type.

**What are the implications for public health practice?**

Providers should be aware of the national guidelines for the treatment of sexually transmitted infections. Monitoring of treatment practices is a critical public health priority to help assure that patients receive the highest quality of care, and to address the emerging threat of antimicrobial-resistant gonorrhea.

## Footnotes

* Case weights developed based on proportion sampled in each jurisdiction; nonresponse adjustments developed by gender and age group. All analyses were conducted using statistical software with linearized Taylor-Series 95% confidence intervals.
